# Overexpression of 2-mercaptoethanesulfonate biosynthesis genes *comDE* protects methane-producing archaea from oxidative stress

**DOI:** 10.1128/jb.00257-25

**Published:** 2025-11-12

**Authors:** Alicia M. Salvi, Connor J. Hines, Nicole R. Buan

**Affiliations:** 1 Department of Biochemistry, Redox Biology Center, University of Nebraska-Lincoln 315569, Lincoln, Nebraska, USA; University of Virginia School of Medicine, Charlottesville, Virginia, USA

**Keywords:** methanogen, archaea, *Methanosarcina*, oxidative stress, 2-mercaptoethanesulfonate, coenzyme M

## Abstract

Coenzyme M (2-mercaptoethane sulfonate, CoM) is an essential low-molecular-weight thiol in methanogenic archaea (methanogens) that serves as a methyl carrier and as a component of CoM-S-S-CoB heterodisulfide comprising CoM and coenzyme B (7-mercaptoheptanoylthreoninephosphate), which serves as the terminal electron acceptor in methanogenesis. Increasing the amount of CoM in *Methanosarcina acetivorans* cells by overexpressing its proposed biosynthesis genes results in faster growth on methanol or methanol + acetate medium in the absence of sulfide. Furthermore, CoM overproduction enhances resistance to oxidative stress in the *ΔhdrABC* mutant genetic background in which the nonessential methylotrophic-specific CoM-S-S-CoB heterodisulfide reductase enzyme, HdrABC, has been deleted. The *ΔhdrABC* mutant is resistant to 5% O_2_ atmosphere, and overexpression of CoM biosynthesis genes resulted in resistance to up to 2 mM hydrogen peroxide in a stress assay. Increased resistance to O_2_ is correlated with 34% lower CoM-SH level in the *ΔhdrABC* and 16% lower CoM-SH level in the *ΔhdrABC com^+^* mutant strains versus the parent strain, while resistance to H_2_O_2_ stress is correlated to a higher ratio of reduced CoM-SH to total CoM in the parent versus the *com^+^* (8.7%), *ΔhdrABC* (10%)*,* and *ΔhdrABC com^+^* (16%) mutant strains. Our study suggests increased expressions of genes encoding coenzyme M biosynthesis, in conjunction with deletion of HdrABC, increase oxidative stress resistance of the strictly anaerobic methanogen *Methanosarcina acetivorans*.

**IMPORTANCE:**

Methanogens are key organisms in the global carbon cycle with potential to be harnessed to produce renewable energy and transportation fuel. Methanogens are strict anaerobes found in subsurface sediment, anaerobic digesters, and digestive tracts of animals such as the rumen. Our results suggest methanogens can adapt to prolonged exposure to oxidative stress under the appropriate environmental conditions, and it may be possible to engineer thiol redox homeostasis and oxidative stress resistance in methanogens. Engineering redox homeostasis and oxidative stress resistance in methanogens and other strict anaerobes has the potential to reduce technical barriers to culturing, thus accelerating research progress on a wide variety of non-model microbes, and ultimately broadening potential biotechnology applications related to sustainable food, fuel, and biomedical uses.

## INTRODUCTION

**Fig 1:**
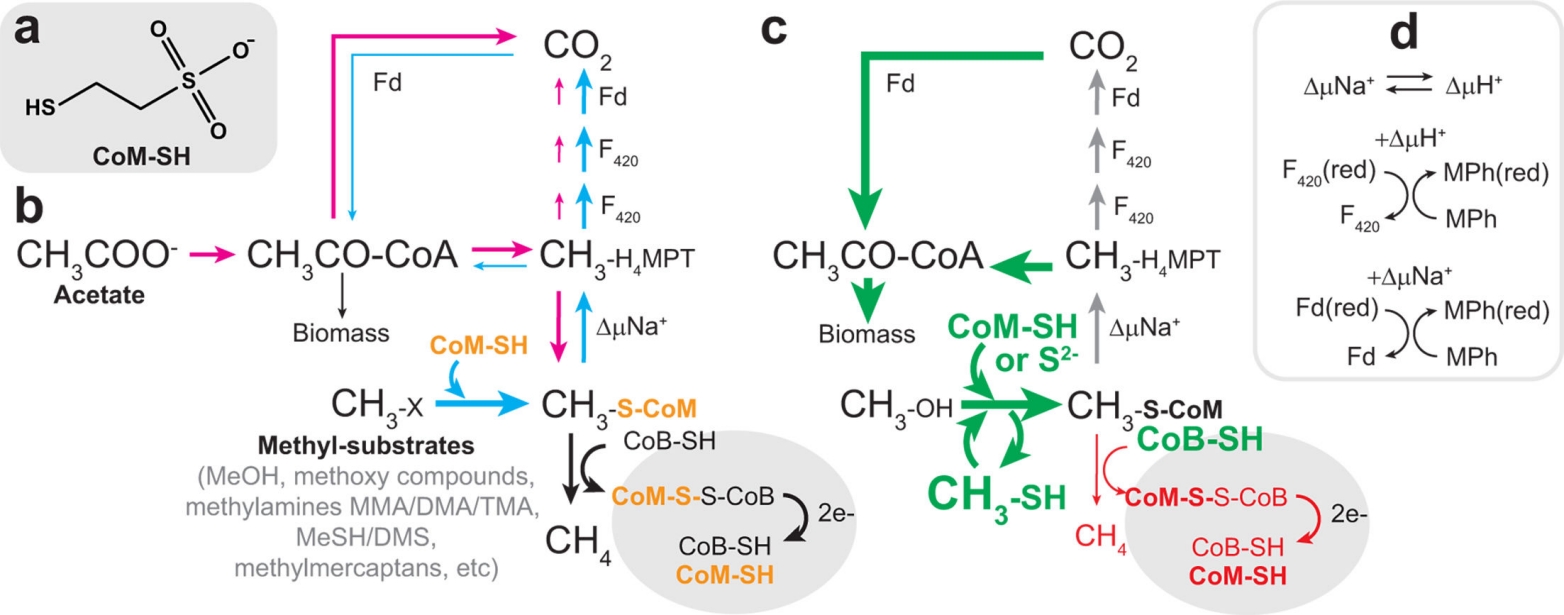
Roles of coenzyme M and Hdr in methanogenesis. (**a**) Structure of Coenzyme M, 2-mercatoethanesulfonate. (**b**) Coenzyme M is essential in methanogenesis, where it serves as a C1-carrier and half of the terminal electron acceptor CoM-S-S-CoB heterodisulfide. Coenzyme M is represented in bold orange text. The methylotrophic methanogenesis pathway is represented in cyan. Acetoclastic methanogenesis is represented in magenta. Reactions common to all methanogenesis pathways are in black arrows. Heterodisulfide reductase, Hdr (shaded oval) reduces the terminal electron acceptor CoM-S-S-CoB using two electrons to regenerate CoM-SH and CoB-SH thiols. (**c**) Effect of *ΔhdrABC* deletion on methyltrophic growth. Green indicates upregulated genes and increased metabolic flux; red indicates decreased mRNA transcripts and metabolic flux; gray arrows indicate unchanged mRNA transcript abundance and metabolic flux. (**d**) Energy conservation reactions in *M. acetivorans*. CH_3_CO-CoA, acetyl coenzyme A; CH_3_-H_4_MPT, methyl tetrahydromethanopterin; CH_3_-S-CoM, methyl coenzyme M; CH_3_-X, methylotrophic substrates such as methanol, methyl sulfides, methylamines, and methoxy compounds; CoB-SH, coenzyme B thiol; CoM-SH, coenzyme M thiol; e-, electrons; Fd, ferredoxin; Fd(red), reduced ferredoxin; F_420_, deazaflavin cofactor F_420_; F_420_(red), reduced F_420_; MPh, methanophenazine; MPh(red), reduced methanophenazine; ΔµH^+^, reaction coupled to a transmembrane proton gradient; ΔµNa^+^, reaction coupled to a transmembrane sodium gradient.

Methanogens are organisms that grow by producing methane gas via the Wolfe cycle of methanogenesis or variations thereof (Fig. 1) (1). They thrive in a variety of anaerobic habitats such as in the deep ocean or subsurface sediment, in digestive tracts of insects and animals, and in anaerobic digesters. They dominate in anaerobic environments where sulfate or other more thermodynamically favorable terminal electron acceptors (such as sulfate) are absent. Recent estimates suggest that ~0.5 Gigatons (Gt) of annual global methane emissions are contributed by methanogens; however, this is an underestimate of total methanogenic activity, which is offset by aerobic and anaerobic methanotrophic microbes (2). It is estimated that approximately 1/3 of biogenic methane is derived from reduction of CO_2_ to methane and 2/3 from acetate via a modified acetoclastic pathway (3). Thus, methanogens play an important role in biogeochemical nutrient cycling and climate (4).

*Methanosarcina acetivorans* was originally isolated from marine sediment (5) and is an emerging model for exploring the biotechnology potential of methanogens to produce renewable fuels and chemicals from inexpensive nonfood feedstocks (for example, methane, CO_2_, CO, formate, and methanol) via a modified Wolfe cycle (6, 7). *M. acetivorans* can naturally grow on methylotrophic substrates (such as methanol, methylamines, and methylsulfides), carbon monoxide, or acetate. It can also participate in interspecies electron transfer (8). Recently, *M. acetivorans* has been engineered to increase the rate of methanogenesis (9, 10), produce high yields of isoprene (11), and has been converted into an acetogen (12) or to grow in the reverse methanotrophic direction (13, 14). Enhancement of these processes through genetic selection and/or genetic engineering requires a detailed understanding of how intracellular redox homeostasis is maintained to ensure efficient functioning of metabolism under changing process conditions. A central molecule in methanogenesis and redox homeostasis is 2-mercaptoethanesulfonate, coenzyme M (CoM).

**Fig 2:**
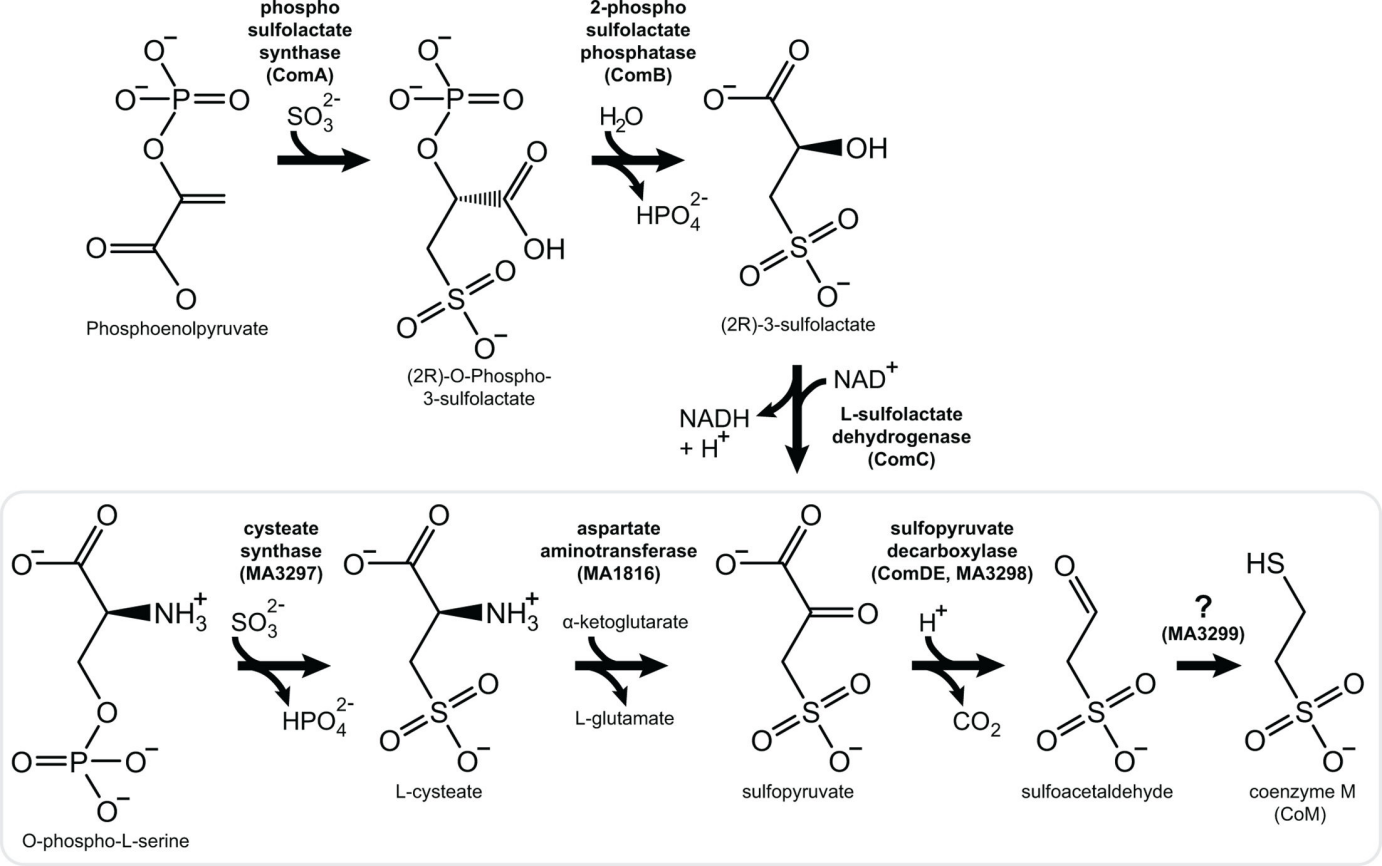
CoM biosynthesis pathways in methanogens. In *Methanosarcinales* and Class II methanogens (gray box), CoM synthesis begins with O-phospho-L-serine. Cysteate synthase (MA3297) catalyzes a β-elimination of phosphate from O-phospho-L-serine. followed by a β-addition of sulfite to produce L-cysteate. Aspartate aminotransferase (MA1816) catalyzes a transamination reaction between L-cysteate and α-ketoglutarate to form sulfopyruvate and L-glutamate. Sulfopyruvate decarboxylase (ComDE, MA3298) enzymatically decarboxylates sulfopyruvate to form sulfoacetaldehyde. Enzymatic conversion from sulfoacetaldehyde to CoM is currently undocumented, although it is believed that a reductive reaction between sulfide and sulfoacetaldehyde could form CoM autocatalytically. Class I methanogens instead synthesize CoM from phosphoenol pyruvate using ComABC enzymes, which are lacking in Class II methanogens.

Coenzyme M is the smallest coenzyme discovered to date. It is essential in methanogens, where it acts as a C1 carrier that accepts methyl groups from corrinoid methyltransferases to produce CH_3_-S-CoM. It is also a component of the CoM-S-S-CoB heterodisulfide formed from coenzyme M and coenzyme B (7-mercaptoheptanoyl threonine phosphate), which serves as the terminal electron acceptor in the methanogenic energy conservation pathway (Fig. 1) (15, 16). CoM is synthesized by at least two pathways in methanogens (Fig. 2) (17). In Methanococcales, Methanobacteriales, and Methanopyrales, phosphoenolpyruvate is converted to sulfoacetaldehyde by ComABCD/E enzymes, while Methanosarcinales and Methanomicrobiales instead synthesize sulfoacetaldehyde from L-phosphoserine using cysteate synthase (*MA3297*), a general aspartate aminotransferase (*aspAT*), and sufopyruvate decarboxylase, *comDE* (*MA3298*). The last step in the pathway is the addition of sulfur and reduction of sulfoacetaldehyde to form CoM, in what is thought to be a non-enzymatic reaction or potentially catalyzed by the neighboring methanogenesis marker protein MA3299 (18–21). CoM is also used for anaerobic alkane metabolism by a few bacteria, which synthesize it from phosphoenolpyruvate in an example of convergent evolution (22, 23).

The Hdr enzyme is necessary to reduce CoM-S-S-CoB to regenerate CoM-SH and CoB-SH thiols for subsequent rounds of methanogenesis (Fig. 1). Hdr comes in two versions in *Methanosarcina*, an essential membrane-bound cytochrome-containing HdrED that conserves energy and a soluble HdrABC that uses electrons from ferredoxin and/or reduced cofactor F420 to reduce CoM-S-S-CoB. HdrA1B1C1 is cotranslated from a single operon and is specific to methylotrophic substrates, while HdrA2C2B2 is essential although preferentially expressed during acetoclastic methanogenesis and is transcribed from two operons, HdrA2:polyferredoxin and HdrC2B2 (24). In previous work, it was observed that when the genes encoding the methylotrophic-specific HdrA1B1C1 enzyme (HdrABC) were deleted, cells were still viable, but ^13^C NMR and transcriptomic studies suggested the *ΔhdrABC* mutant phenotype was caused by decreased ferredoxin redox cycling and changes in coenzyme M (CoM-SH) homeostasis (24). Deletion of genes encoding the methylotrophic HdrABC resulted in upregulation of methyltransferases, carbon monoxide dehydrogenase CdhA2, sulfonate transporters, and genes proposed to be involved in CoB-SH synthesis (2-isopropylmalate synthase, *MA4615*) and CoM-SH synthesis (cysteate synthase and sulfopyruvate decarboxylase *comDE*, *MA3297-3298*) (24). These data were interpreted to suggest that in the absence of HdrABC, reduction of CoM-S-S-CoB is slowed, resulting in slower uptake of the substrate by methyltransferases, resulting in production of methane thiol (MeSH) and dimethylsulfide (DMS), which can ultimately be used as substrates (Fig. 1c). As a result, while the kinetics of methanogenesis are decreased, CoM-SH flux is increased to compensate, and metabolic efficiency is increased (9, 24). This model was further supported when it was shown that adding sulfide or supplementing cultures with acetate and exogenous CoM-SH can also partially rescue the *ΔhdrABC* mutant growth defect (25). These findings suggest that intracellular CoM-SH pools and redox homeostasis can be altered in *M. acetivorans*. Therefore, we wanted to test if directly increasing the intracellular CoM-SH pool in cells by overexpressing the *com* locus (*MA3296-MA3298*) can affect growth and redox homeostasis.

## MATERIALS AND METHODS

### Culture conditions

Organisms were obtained from the sources listed in Table 1. Methanogens were grown in high salt mineral medium (HS) (200 mM NaCl, 45 mM NaHCO_3_, 13 mM KCl, 54 mM MgCl_2_•6H_2_O, 2 mM CaCl_2_•2H_2_O, 2 µM 0.1% resazurin (w v^−1^), 5 mM KH_2_PO_4_, 19 mM NH_4_Cl, 2.8 mM cysteine•HCl, 0.1 mM Na_2_S•9H_2_O, trace elements, and vitamin solution) as described (26) and supplemented with a carbon and energy source (methanol, 125 mM; trimethylamine, 50 mM; sodium acetate, 120 mM) and 2 mg L^−1^ puromycin, as needed at 35°C. For growth on solid media, 1.4% agar was added to HS media. Methanogens were grown anaerobically in a custom B-type Coy anoxic chamber (Coy Labs, Grass Lake, MI) under a 5% H_2_/20% CO_2_/75% N_2_ (± 3%) (Matheson Gas, Lincoln, NE) atmosphere. Cells incubated outside of the anaerobic chamber are contained in glass Balch tubes secured with butyl rubber stoppers (Bellco Glass, Vineland, NJ) and aluminum crimps (Wheaton, Millville, NJ).

**TABLE 1:** Primers, plasmids, and strains used in this study

Primers			
Name	Sequence (5’→3’)	Purpose	Source
oNB52	GAAGCTTCCCCTTGACCAAT	ϕC31 screen-all#1; validation of plasmid integration	(27)
oNB53	TTGATTCGGATACCCTGAGC	ϕC31 screen-C2A#1; validation of plasmid integration	(27)
oNB54	GCAAAGAAAAGCCAGTATGGA	ϕC31 screen-pJK200#1; validation of plasmid integration	(27)
oNB55	TTTTTCGTCTCAGCCAATCC	ϕC31 screen-pJK200#2; validation of plasmid integration	(27)
oNB95	aaaaaaaaaaaaggcgcgccTTCCGCATTTTGGACAGACGAAA	Amplifies AscI P-*MA3296-8* fwd	This study
oNB96	aaaaaaaaaaaaggatccGAGATCCTTTGCGCTTTTCTACGAAA	Amplifies BamHI P*-MA3296-8* rev	This study
oNB98	ACCTCTTACCGTGCATATGTCTTGAGTTTAG	Amplifies upstream of *MA3298* rev	This study
oNB99	AACGAAATTTTTCGTAGAAAAGCGCAAAGGA	Amplifies downstream of *MA3298* fwd	This study
oNB103	aaaaaaaaaaaacatATGTACGTGGTAAACCCGGAAGAAAAAGT	NdeI *MA3298* fwd	This study
oNB104	aaaaaaaaaaaaggatccGAGATCCTTTGCGCTTTTCTACGAAA	*MA3298* BamHI rev	This study
oNB121	GCACCCAGGCACATTGTTC	*hdrA* 301 rev	(9)
oNB122	TACTGGGGTTTCTGGGAGAC	*hdrA* 1024 rev	(9)
oNB123	ATGCCCTCTCCGTAAATGAG	*hdrA* 1880 fwd	(9)
oNB124	GATTCAAGCACACTGCGATC	*hdrC* 2616 rev	(9)
oNB376	ATGGTCTTGCTCTCAGCGATGA	Identify *hdrABC* deletion: amplifies 5′ upstream of *MA3128*	(9)
oNB377	AGGTGTTGGTATGAAAATCAGCAAGG	Identify *hdrABC* deletion: amplifies 3′ downstream of *MA3126*	(9)

Plasmids			
Plasmid name	Genotype		Reference
pJK026A	*attB cat pac hpt uidA*		(27)
pJK027A	*attB cat pac hpt uidA*		(27)
pNB710	pJK027A:NdeI *MA3298* BamHI *P_tet_* promoter		This study
pNB711	pJK026A:AscI *MA3296-8* BamHI native promoter		This study
pNB714	pJK026A:Nde *MA3298* BamHI *P_mcr_* promoter		This study

Organisms			
NB#	Organism	Genotype	Reference
*Escherichia coli* strains			
3	DH5α F’ *lacI^q^*	F´ *proA^+^B^+^ lacI^q^ ∆(lacZ)M15 zzf::Tn10 (TetR*) / *fhuA2∆(argF-lacZ)U169 phoA glnV44 Φ80Δ(lacZ)M15 gyrA96 recA1 endA1 thi-1 hsdR17*	New England Biolabs
10	DH10B *att*::pAMG27	F- *mcrA Δ(mrr-hsdRMS-mcrBC) φ80lacZΔM15 ΔlacΧ74 recA1 araD139 Δ(ara-leu) 7697 galU galK rpsL (StrR) endA1 nupG λ- att::Prha trfA33-254D*	(27)
55	DH10B *att*::pAMG27/pNB710	F- *mcrA Δ(mrr-hsdRMS-mcrBC) φ80lacZΔM15 ΔlacΧ74 recA1 araD139 Δ(ara-leu) 7697 galU galK rpsL (StrR) endA1 nupG λ- att::Prha trfA33-254D*/ pNB710	This study
58	DH10B *att*::pAMG27/pNB711	F- *mcrA Δ(mrr-hsdRMS-mcrBC) φ80lacZΔM15 ΔlacΧ74 recA1 araD139 Δ(ara-leu) 7697 galU galK rpsL (StrR) endA1 nupG λ- att::Prha trfA33-254D*/ pNB711	This study
84	DH10B *att*::pAMG27/pNB714	F- *mcrA Δ(mrr-hsdRMS-mcrBC) φ80lacZΔM15 ΔlacΧ74 recA1 araD139 Δ(ara-leu) 7697 galU galK rpsL (StrR) endA1 nupG λ- att::Prha trfA33-254D*/pNB714	This study
*Methanosarcina acetivorans* C2A strains			
34	*parent*	*Δhpt::ϕC31 int attP*	(27)
36	*ΔhdrABC*	*Δhpt::ϕC31 int attP ΔhdrA1B1C1*	(24)
111	*P_tet_comDE^+^*	*Δhpt::ϕC31 int att:pNB710*	This study
112	*ΔhdrABC P_tet_comDE^+^*	*Δhpt::ϕC31 int att:pNB710 ΔhdrA1B1C1*	This study
114	*com^+^*	*Δhpt::ϕC31 int att:pNB711*	This study
115	*ΔhdrABC com^+^*	*Δhpt::ϕC31 int att:pNB711 ΔhdrA1B1C1*	This study
117	*P_mcr_comDE^+^*	*Δhpt::ϕC31 int att:pNB714*	This study
118	*ΔhdrABC P_mcr_comDE^+^*	*Δhpt::ϕC31 int att:pNB714 ΔhdrA1B1C1*	This study

*Escherichia coli* cells were grown aerobically in 0.5% glucose Lysis Broth (LB) (28) with shaking at 37°C with supplementation as appropriate: 0.5% agar, rhamnose (1 mM), chloramphenicol, 10–35 µg mL^−1^). Chemicals and reagents were sourced from Millipore Sigma (St. Louis, MO) or Fisher Scientific (Waltham, MA).

Culture growth was measured using a Spectronic D spectrophotometer (ThermoFisher, Waltham, MA) fitted with a Balch tube (18 mm) modification or using a Tecan Sunrise UV/Vis spectrophotometric plate reader (Tecan, Männedorf, Switzerland). Population doubling times were calculated for each individual biological replicate by solving for the linear slope (r^2^ >0.95) of the natural log transform of the culture optical density measured at 600 nm and averaged together by treatment.

### Plasmid cloning, strain construction, and validation

Plasmids and primers shown in Table 1 were designed using VectorNTI software (ThermoScientific, Waltham MA). PCR primers were synthesized by Integrated DNA Technologies (IDT, Coralville, IA). The proofreading Phusion Flash PCR Master Mix was used for all PCR amplification procedures (ThermoScientific, Waltham, MA). Promega Wizard SV Gel and PCR Clean-up kits (Madison, WI) were used for DNA purification. Fast Digest Restriction Enzymes (*BamHI* and *NdeI*) were purchased from ThermoScientific (Waltham, MA). AscI was purchased from NEB (Ipswich, MA). DNA fragments were assembled using the Sequence and Ligation Independent Cloning (SLIC) protocol previously described (29). Two promoters, *P_mcr_* and *P_tet_*, were used to test whether promoter strength affected the observed phenotypes when the *com* locus (MA3296-MA3298) or the *comDE* gene alone (MA3298) was overexpressed (Table 2). *P_mcr_* is a strong constitutive promoter. *P_tet_* is identical to *P_mcr,_* except it contains a *tetO1* TetR repressor binding site, resulting in lower constitutive expression in *tetR*-deficient strains. All plasmid inserts were verified by sequencing (Eurofins, Louisville, KY).

**TABLE 2:** Genes overexpressed in this study

MA#	Location	Gene ID	Predicted function
MA3296	4068167–4068631	MA_RS17195	Hypothetical
MA3297	4068958–4070208	MA_RS17200	Cysteate synthase
MA3298	4070349–4071512	*comDE*	Sulfopyruvate decarboxylase, beta

**Fig 3:**
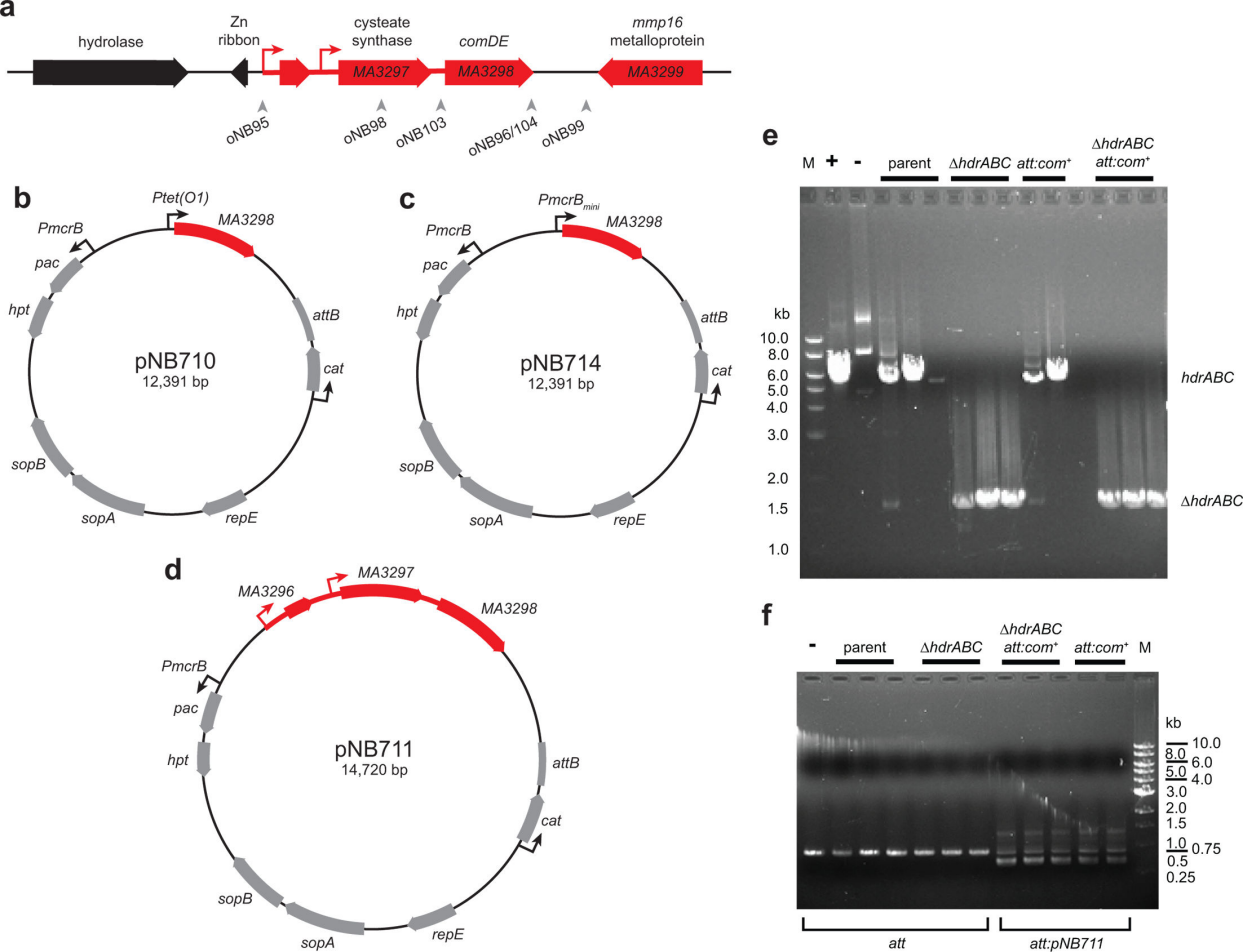
Plasmids and strain validation. (**a**) The putative *com* genetic locus in *M. acetivorans* (red). Oligonucleotide primers used for cloning and strain validation are shown by gray arrows. (**b**) Plasmid map for pNB710 resulting in constitutive or tetracycline-inducible overexpression of *comDE* (*MA3298*). (**c**) Plasmid map for pNB714 resulting in high constitutive overexpression of *comDE*. (**d**) Plasmid map for pNB711, which increases the *com* locus copy number when introduced into host strains. (**e**) Validation of *ΔhdrABC* deletion by PCR screen. (**f**) Validation of plasmid pNB711 integration by PCR screen. All primers are listed in Table 1. +, positive control; −, negative control; kb, kilobase; M, DNA marker.

After growth curve data were collected, strain genotypes were confirmed using a PCR assay as previously described using the primers listed in Table 1 (9, 27). To verify deletion of the *hdrA1B1C1* operon (*MA3126–MA3128*), primers oNB376 and oNB377 were used to amplify a diagnostic 1.607 kp fragment (Fig. 3e). To verify transformation and plasmid integration on the genome, a four-primer screen was used (Fig. 3f). Untransformed genomes yield one band at 910 bp (*attP* amplified by primers oNB52 and oNB53), strains transformed with pNB711 in which the plasmid has integrated at the *att* recombination site produce diagnostic bands at 680 bp (*attR* amplified by primers oNB52 and oNB55) and 741 bp (*attL* amplified by primers oNB53 and oNB54), double-integrants and circular plasmid yield a band at 511 bp (*attL* amplified by primers oNB54 and oNB55), and a weak nonspecific band at approximately 1.479 kb likely amplified by primers oNB53 (5’NNGANNCGGATACCCNNNNN) and oNB55.

### Oxidative stress assays

Oxidative stress assays were carried out as described (30, 31) after adapting *M. acetivorans* to the HS methanol medium without resazurin or sodium sulfide for 15 generations (three passages of 0.25 mL into 10 mL cultures). For O_2_ stress assays, cells were grown to an OD_600_ of 0.4, whereupon cultures were injected with either sterile 100% O_2_ gas (Matheson) to 1% or 5% vol/vol/ headspace at 1 atm, ambient air (20% O_2_/80% N_2_) at 1 atm, or freshly obtained H_2_O_2_ at 1.5 mM or 3 mM final concentration.

### Thiol extraction and quantification

*M. acetivorans* was grown in 10 mL HS media with MeOH as the carbon source at 35°C until OD_600_ = 0.5–0.6 (mid exponential). Cultures were centrifuged anaerobically, and cells were washed twice with 0.85 M bicarbonate-buffered sucrose. Cells were lysed by osmotic shock by resuspension in 1 mL anaerobic H_2_O, and low-molecular-weight thiols were extracted and derivatized with monobromobomane (mBBr). The derivatization of thiol compounds with monobromobimane (mBBr) was modified based on published methods (32, 33). Because of its small size and negative charge, CoM-SH must be derivatized to allow quantification. However, because it is a reactive thiol, CoM-SH may become oxidized in the cell due to metabolism or inadvertently during extraction, and a portion of the CoM sample may be in the oxidized CoM-S-R form, where “R” represents any of the following: CoM-S-CH_3_, CoM-S-S-CoM, CoM-S-S-CoB, CoM-S-Cys (free cysteine or a protein thiol), CoM-S-Fe(II)/S, CoM-S-Co(II)rrinoid, CoM-S-Fe(II)heme, and CoM-S-Ni(II)F430. CoM-S-Ni(II)F430 is an obligate intermediate in the methyl-coenzyme M reductase reaction, and although CoM-S-Co(II)rrinoid has not been described in literature to our knowledge, corrinoids react readily with reduced glutathione, cysteine, and other thiols to produce thiolato-S-Co(II)rrinoids (34–37). To quantify free CoM-SH vs total CoM (CoM-SH + CoM S-R), matched samples were split: one was derivatized with mBBR directly to quantify free CoM-SH, while the other matched sample was reduced with KBH_4_ before derivatization with mBBr to quantify total CoM (CoM-SH + CoM S-R). The concentration of CoM-S-R was calculated by subtracting the CoM-SH amount from the total CoM measured after KBH_4_ reduction. Briefly, after removing an aliquot for protein quantification (Bradford), half the extracts were reduced with 92.6 mM KBH_4,_ while the other half was diluted with H_2_O. Unreacted KBH_4_ was quenched with 2.5 μL 1M HCl, followed by 2.5 μL 1M NaOH. Samples were diluted with 615 mL buffer (200 mM HEPES, 5 mM diethylenetriamine pentaacetate (DTPA) pH 8.2) reacted with 10 μL 20 mM mBBr in acetonitrile in the dark for 30 minutes. The derivatization reaction was quenched with 100 mL methanesulfonic acid. Derivatized thiols were quantified in the Nebraska Center for Biotechnology core facility by reverse-phase high-pressure liquid chromatography (HPLC) using either a Dionex UltiMate 3000 HPLC with diode array and fluorescence detection (Thermo Scientific, Waltham, MA) or Agilent Technologies 1290 Infinity II HPLC with fluorescence detection (Agilent Technologies, Santa Clara, CA). Samples were injected onto a Supelcosil LC-18 15 cm by 4.6 cm, 5 µm column fitted with a SupelGuard C18 guard column (Sigma-Aldrich). Analytes were separated by gradient from 10% ACN, 0.1% TFA (vol/vol) in H_2_O to 99% ACN, 0.1% TFA (vol/vol) mobile phase at 1 mL min^−1^ and washed with 100 % methanol at 2.5 mL min^−1^ (38). mBBr was followed by 380 nm (ex) and 470 nm (em). Peak areas were normalized to soluble protein concentrations and quantified by comparison to standards: cysteine (Fisher), coenzyme M (Fisher), and coenzyme B (Buan Lab stock synthesized as described) (39, 40).

## RESULTS

### Construction of *com*^*+*^ overexpression strains

To uncouple CoM-SH synthesis from direct or indirect effects of the *ΔhdrABC* mutation, we synthesized several plasmids in an attempt to influence intracellular CoM-SH levels. Unfortunately, the CoM-SH biosynthetic pathway is not fully understood in *M. acetivorans*, and it seems the pathway differs from other methanogens as no clear homologs for several steps in the pathway are identified (Fig. 2). Therefore, we focused on the two genes that appear to be upregulated in the *ΔhdrABC* mutant (Fig. 1c), *MA3297* (cysteate synthase), and *MA3298* (*comDE*) (Fig. 3a). We cloned MA3298, encoding *comDE*, into pJK026A and pJK027A, resulting in plasmids pNB710 (Fig. 3b) and pNB714 (Fig. 3c), respectively. pNB710 expresses *comDE* from a *Ptet(01*) promoter (medium-strength), and pNB714 expresses *comDE* from the highly expressed constitutive *PmcrB_mini_* promoter. We also replaced the promoter of pJK027A with the entire MA3296-MA3298 operon and upstream promoter region to produce pNB711 (Fig. 3d). All three plasmids contain ϕC31 *attB* sites to integrate onto the parent strain chromosome. Each plasmid was transformed into the parent strain and the *ΔhdrABC* mutant, where genotypes were confirmed using PCR screens (Fig. 3e and f) using primers listed in Table 1.

### Overexpression of *P*_*mcr*_*comDE*^*+*^ improves growth on methanol and methanol + acetate as energy sources

**Fig 4:**
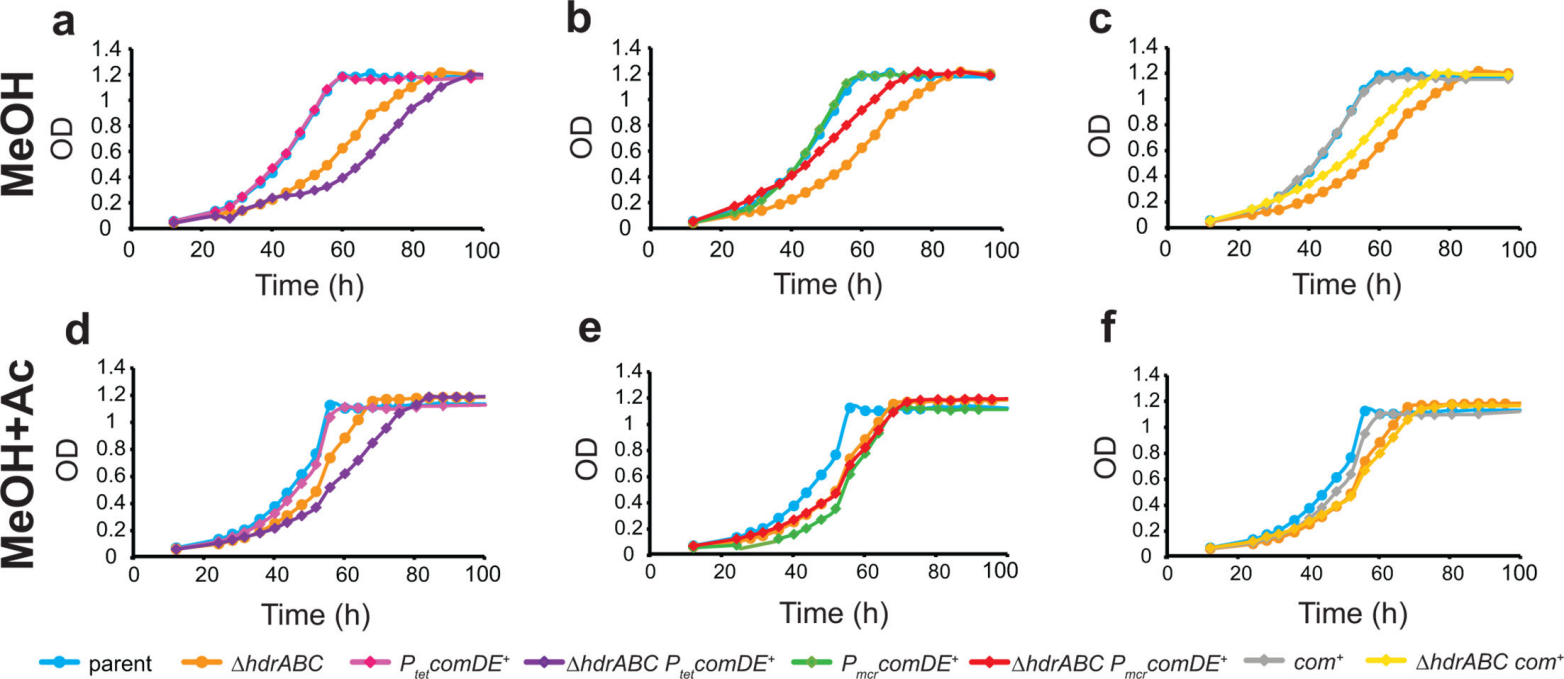
Effect of *com* overexpression on growth of *M. acetivorans* on methanol and methanol + acetate. (**a–c**) Methylotrophic growth curves on 125 mM methanol. (**d–f**) Mixotrophic growth curves on 125 mM methanol plus 40 mM acetate. (**a and d**) Comparison of parent versus *ΔhdrABC* strains overexpressing *P_tet_comDE^+^*. (**b and e**) Parent versus *ΔhdrABC* strains overexpressing *P_mcr_comDE^+^*. (**c and f**) Parent versus *ΔhdrABC* strains overexpressing *com^+^*. Parent and *ΔhdrABC* curves are the same in each panel for comparison. Error bars have been omitted for clarity. Each data point represents the average of at least four biological replicates. OD, optical density at 600 nm.

Strains were grown on methanol or methanol +acetate as energy sources to determine if the overexpression of *com* genes affected growth during methylotrophic or mixotrophic growth. Growth on acetate as the sole energy source was not tested because previous work showed the parent and *ΔhdrABC* mutant strains had the same growth rates on this substrate. On methanol, overexpression of *P_mcr_comDE^+^* resulted in a slightly decreased population doubling time to 8.97 h (±0.233) versus the parent at 10.27 h (±0.518) (Table 3). None of the plasmids affected growth rates of the *ΔhdrABC* mutant on methanol; however, differences in lag times were observed (Fig. 4a through c). Mixotrophic growth on methanol + acetate as energy sources was tested (Fig. 4d through f) as previous research showed addition of exogenous CoM-SH under these conditions does not affect the parent strain but is able to rescue the growth rate defect of the *ΔhdrABC* mutant strain (25). Under mixotrophic conditions, both the *P_mcr_comDE^+^* and the *com^+^* overexpression plasmids resulted in faster growth rate when transformed into the parent strain (8.22 h ± 0.196 and 9.68 h ± 0.323 versus 10.81 h ± 0.139, respectively). None of the overexpression plasmids affected the growth rate of the *ΔhdrABC* mutant, indicating that overexpression of *comDE* alone or of the entire *com* locus was not capable of producing enough CoM-SH to overcome the lack of HdrABC.

**TABLE 3:** Growth rates on methanol or methanol + acetate as energy sources^*a*^

Strain	Methanol				Methanol + acetate			
	Doubling time (h)	Std dev	*P* vs parent	*P* vs *ΔhdrABC*	Doubling time (h)	Std dev	*P* vs parent	*P* vs *ΔhdrABC*
Parent	10.27	0.518	1	0.0190	10.81	0.139	1	0.0001
*P_tet_comDE^+^*	10.31	0.421	NS	0.0171	10.76	0.142	NS	0.0001
*P_mcr_comDE^+^*	8.97	0.233	0.0032	0.0005	8.22	0.196	2E-08	2E-08
*com^+^*	10.15	0.590	NS	0.0088	9.68	0.323	0.0002	7E-06
*ΔhdrABC*	12.64	1.010	0.0190	1	11.79	0.258	0.0001	1
*ΔhdrABC P_tet_comDE^+^*	15.39	1.651	0.0012	NS	13.19	1.446	0.0145	NS
*ΔhdrABC P_mcr_comDE^+^*	15.07	0.738	0.0004	NS	11.95	0.349	0.0006	NS
*ΔhdrABC com^+^*	15.90	2.924	0.0166	NS	12.84	1.516	0.0284	NS

^*a*^ NS, not significant (*P* > 0.05). Data were obtained from at least four biological replicates.

### Overproduction of CoM results in increased resistance to oxidative stress

As a low-molecular-weight thiol, CoM-SH, is often compared to glutathione (GSH) as both are present at low mM concentration (~2 mM in methanogens and ~3 mM in bacterial or eukaryal cells, respectively) (41–44). Because methanogens do not synthesize GSH (45), it has been hypothesized that perhaps CoM-SH, in addition to playing its vital roles in methanogenesis (Fig. 1), may also be involved in resistance to oxidative stress by virtue of its inherent chemical properties as a thiol molecule. Recently, increased CoM-SH production was observed to occur as a result of adaptation to exposure to oxygen and heavy metals (46, 47). We wanted to test whether the strains we generated may also be tolerant to oxidative stress.

**Fig 5:**
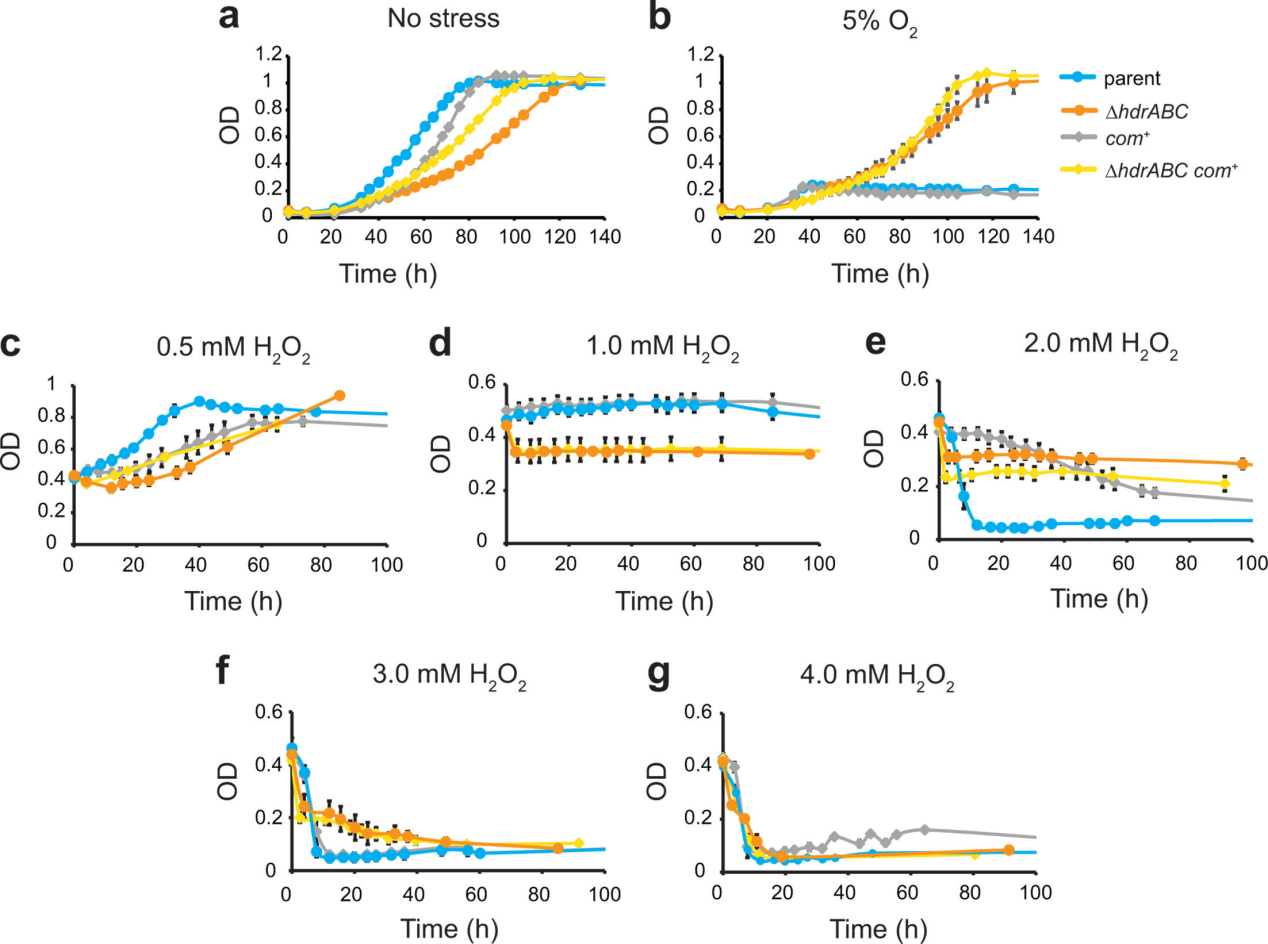
Deletion of *hdrABC* and overproduction of CoM results in increased resistance to oxidative stress. (**a**) Growth of strains in medium with methanol as the sole energy source and sulfide omitted. Error bars are removed for clarity. Data for parent (blue) and *ΔhdrABC* (orange) strains in (**a**) are graphed from Salvi et al. as part of the same experiment for comparison.(25) Subsequent panels show growth of strains in the same medium. (**b**) Growth of each strain when culture headspace contained 5% O_2_. (**c–g**) Cultures were grown to OD = 0.4 under unstressed conditions as in (**a**), and then fresh H_2_O_2_ to the indicated concentration was added at time 0 h. Each data point represents the average of at least four biological replicates. Error bars indicate standard deviation. OD, optical density at 600 nm.

The parent, *ΔhdrABC, P_mcr_comDE^+^, and com^+^* strains were adapted to methanol medium without added sulfide, which serves as a chemical antioxidant. Under these conditions, without stress (Fig. 5a), we observed slightly increased growth rates of the *P_mcr_comDE^+^ and com^+^* strains in both the parent and *ΔhdrABC* genetic backgrounds (Table 4). Under O_2_ stress, in which cultures are inoculated into tubes with a headspace atmosphere containing 5% O_2_, we were surprised to observe the *ΔhdrABC* and *ΔhdrABC com^+^* strains are very resistant and are ultimately able to reach full culture density (Fig. 5b). Under these stress conditions, the *ΔhdrABC* mutant had a doubling time of 26.14 h (±3.147) and the *ΔhdrABC com^+^* mutant had a doubling time of 15.30 h (±1.713, *P* = 0.006 vs *ΔhdrABC*). In contrast, the parent and *com^+^* strains did not achieve an OD higher than 0.2. These results are interpreted to suggest that the *ΔhdrABC* mutation results in changes to cellular physiology that allows cells to detoxify molecular oxygen and that overexpression of *com* genes enhances this effect. Possible mechanisms include increased expression of corrinoid proteins, which are highly sensitive to oxidation and could theoretically directly scavenge oxidants, requiring ATP-dependent repair by the *ram* system (48, 49). Alternatively, corrinoid proteins may nonspecifically react with CoM-S-S-CoM disulfide, forming CoM-SH and CoM-S-corrinoid adducts (which may be regenerated by corrinoid methyltransferases), thus increasing overall CoM-SH turnover as overexpression of the *com* genes alone in the parent background is not sufficient to result in detectable resistance to O_2_.

**TABLE 4:** Growth rates in medium with methanol as the sole energy source without added sulfide^*a*^

Strain	Doubling time (h)	Std dev	*P* vs parent	*P* vs Δ*hdrABC*
Parent	9.64	0.464	1	0.0165
*P_mcr_comDE^+^*	8.96	0.231	NS	0.0034
*com^+^*	9.37	0.689	NS	0.0224
*ΔhdrABC*	10.71	0.336	0.0165	1
*ΔhdrABC P_mcr_comDE^+^*	9.89	0.252	NS	0.0302
*ΔhdrABC com^+^*	12.26	0.771	0.0128	NS

^*a*^ NS, not significant (*P* > 0.05). Data were obtained from at least three biological replicates. Data for parent and *ΔhdrABC* strains are from Salvi et al. as part of the same experiment for comparison (25).

We next tested whether the parent, *ΔhdrABC, com^+^,* and *ΔhdrABC com^+^* strains are resistant to H_2_O_2_ exposure. For these experiments, we focused on strains where *hdrA1B1C1* and *com* genes were expressed from their respective native promoters, mimicking a natural duplication event, and we did not compare *P_mcr_comDE^+^* and *P_tet_comDE^+^* strains in which *com* genes are regulated by different promoters. Cells were grown to the mid-exponential phase (OD = 0.4) and dosed with increasing levels of hydrogen peroxide from 0.5 to 4 mM (Fig. 5c through g). At 1.0 mM H_2_O_2_, the parent and *com^+^* cease growth, while cultures of *ΔhdrABC and ΔhdrABC com^+^* strains showed a slight decrease in optical density and then remained static at a lower final optical density than the parent and *com^+^* strains. Surprisingly, at 2.0 mM H_2_O_2_, the parent strain rapidly lysed within 12 hours, while the *ΔhdrABC, com^+^, and ΔhdrABC com^+^* strains were resistant to lysis. By 4.0 mM H_2_O_2_, cells from all four strains were sensitive to killing. These results indicate that while the *ΔhdrABC* mutant is resistant to 2–3 mM H_2_O_2_ (Fig. 5e and g), adding the *com^+^* overexpression plasmid confers additional resistance at 0.5 mM H_2_O_2_ but not at higher levels of peroxide stress. In the parent genetic background, the *com^+^* overexpression plasmid was only observed to increase resistance to 2.0 mM H_2_O_2_ but not at lower or higher levels of peroxide stress.

### Deletion of *ΔhdrABC* and overexpression of *com* results in increased proportion of reduced CoM-SH

**Fig 6:**
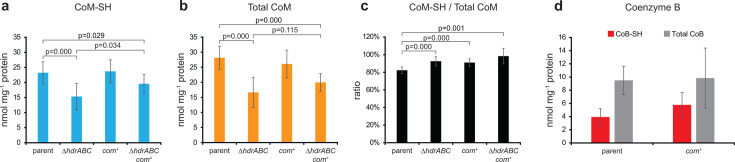
Overexpression of *com* genes results in changes to thiol pools in MeOH-grown cells. (**a**) Quantification of reduced CoM-SH and oxidized CoM (**b**) and from parent (*n* = 12), *ΔhdrABC* (*n* = 9)*, com^+^* (*n* = 10)*,* and *ΔhdrABC com^+^* (*n* = 8) strains. (**c**) Ratio of CoM-SH vs total CoM. Error bars indicate standard deviation. (**d**) Quantification of free CoB-SH and total CoB from parent (*n* = 7) and *com^+^* (*n* = 5) strains. CoB could not be accurately quantified from *ΔhdrABC* and *ΔhdrABC com ^+^* strains. *P* values indicated were calculated by two-tailed T test versus parent strain.

To test whether resistance to oxidative stress is correlated to increased intracellular levels of CoM-SH, we extracted and measured CoM-SH levels in parent, *ΔhdrABC*, *com^+^,* and *ΔhdrABC com^+^* strains (Fig. 6; Table 5). The concentration of free CoM-SH was measured as 23.24 nmol mg^−1^ protein (±3.70) in the parent strain, 15.35 nmol mg^−1^ protein (±4.33) in the *ΔhdrABC* mutant, 23.69 nmol mg^−1^ protein (±3.82) in the *com^+^* mutant, and + nmol mg^−1^ protein (±3.10) in the *ΔhdrABC com^+^* strain (Fig. 6a). Total CoM was derived from the aliquots reduced using KBH_4_ prior to derivatization with mBBr; this quantity non-exhaustively includes CoM-SH, CH_3_-CoM, CoM homodisulfides, and CoM heterodisulfides. The concentration of total CoM was found to be 28.16 nmol mg^−1^ protein (±3.81) in the parent strain, 16.66 nmol mg^−1^ protein (±4.97) in the *ΔhdrABC* mutant, 26.11 nmol mg^−1^ protein (±4.59) in the *com^+^* mutant, and 19.95 nmol mg^−1^ protein (±2.91) *ΔhdrABC com^+^* strain (Fig. 6b). We noted the *ΔhdrABC* strain has a 66% decrease in the amount of free CoM-SH in cell extracts versus the parent strain, confirming previous work that suggested CoM was limiting in the *ΔhdrABC* strain (24). The decreased amount of free CoM-SH and total CoM was partially complemented by *com^+^* expression, confirming a role for MA3297-3298 in CoM biosynthesis (Fig. 6a and b). However, *com^+^* expression in the parent strain did not increase free CoM-SH or total CoM levels, suggesting that *com* gene dosage is not limiting in the parent genetic background. Expression of *com^+^* resulted in higher ratio of CoM-SH to CoM-S-R (oxidized CoM which includes CoM-S-S-B, CoM-S-S-CoM, CoM-S-S-Cys as free amino acid or on a protein, CoM-S-Fe(II)/S, CoM-S-Co(II)rrinoid, CoM-S-Fe(II)heme, or CoM-S-Ni(II)F430 that may be reduced by borohydride, <−1.24V vs SHE) when expressed from the parent background (10.55% increase, *P* = 0.000) and from the *ΔhdrABC* background 6.28% fold increase, *P* = 0.058) (Fig. 6c).

**TABLE 5:** Thiol quantification from MeOH-grown cells (nmol mg^−1^)^*a*^

Strain	Parent	Δ*hdrABC*	*com* ^*+*^	Δ*hdrABC com^+^*
Free CoM-SH				
Avg	23.24	15.35	23.69	19.58
Std dev	3.70	4.33	3.82	3.10
*P* vs parent	1	0.000	NS (0.787)	0.029
*P* vs Δ*hdrABC*	0.000	1	0.000	0.034
Total CoM				
Avg	28.16	16.66	26.11	19.95
Std dev	3.81	4.97	4.59	2.91
*P* vs parent	1	0.000	NS (0.273)	0.000
*P* vs Δ*hdrABC*	0.000	1	0.001	NS (0.115)
Ratio (% CoM-SH/total CoM)				
Avg	82.33%	92.56%	91.02%	98.37%
Std dev	3.74	5.29	4.58	8.82
*P* vs parent	1	0.000	0.000	0.001
*P* vs Δ*hdrABC*	0.000	1	NS (0.511)	NS (0.133)
Free CoB-SH				
Avg	3.94	Not measurable	5.79	Not measurable
Std dev	1.25	–^*b*^	1.87	–
*P* vs parent	1	–	NS (0.101)	–
Total CoB				
Avg	9.50	Not measurable	9.85	Not measurable
Std dev	2.15	–	4.55	–
*P* vs parent	1	–	NS (0.880)	–
Ratio (% CoB-SH/total CoB)				
Avg	43.01	–	74.22	–
Std dev	16.43	–	52.45	–
*P* vs parent	1	–	NS (0.260)	–

^*a*^ CoM was quantified using a standard curve with authentic mBBr-derivatized CoM. Data were obtained from at least eight biological replicates. NS, not significant (*P* > 0.05). CoB was identified by comparison to synthesized CoB and quantified versus the standard curve generated with authentic mBBr-derivatized CoM (9). NS, not significant (*P* > 0.05).

^*b*^ – indicates that data could not be calculated.

The average concentration of free CoB-SH was 3.95 (±1.25) nmol mg^−1^ protein and 5.79 (±1.87) nmol mg^−1^ for the parent (*n* = 7) and *com^+^* (*n* = 5) strains, respectively (*P* = 0.10) (Fig. 6d; Table 5). Total CoB was measured as 9.50 (±2.15) nmol mg^−1^ protein in the parent (*n* = 7) and 9.85 (±4.55) nmol mg^−1^ in the *com^+^* (*n* = 5) strains (*P* = 0.88). CoB could not be reliably quantified in *ΔhdrABC and ΔhdrABC com^+^* extracts, indicating that the concentrations of CoB-SH and total CoB were below the limit of detection. Even though the CoM concentration was elevated in *ΔhdrABC com^+^* compared to CoM level in the *ΔhdrABC* strain, no such increase for CoB was observed. These data suggest overall lower levels of CoB biosynthesis when *hdrABC* is deleted. Additionally, the difference in CoB concentration between parent and *com^+^* strains was not significant, confirming that CoB production was not impacted with the introduction of *com^+^*.

Overall, we observed that the *ΔhdrABC, com^+^,* and *ΔhdrABC com^+^* mutant strains have higher ratios of reduced CoM-SH versus total CoM than the parent strain (Fig. 6c). The increased ratio of free CoM-SH in *ΔhdrABC, com^+,^* and *ΔhdrABC com^+^* strains correlates with resistance to H_2_O_2_ stress, congruent with its role as an antioxidant. The decrease in total CoM in *ΔhdrABC* and *ΔhdrABC com^+^* strains correlates with their increased doubling times, while the increase in CoM-SH and total CoM between the two strains confirms that *com^+^* overexpression improves CoM production in the *ΔhdrABC* genetic background. The absence of increased CoM production in the *com^+^* strain indicates that *com^+^* alone is insufficient in increasing CoM production in the parent genetic background.

## DISCUSSION

Our results support the hypothesis that in addition to serving essential roles as C-1 carrier and part of the terminal electron acceptor, CoM-SH can function as a scavenging antioxidant in *Methanosarcina*. Unlike most bacteria, eukarya, or archaea, methanogens do not produce glutathione or gamma-glutamyl cysteine to use as a general protectant from oxidative stress (45). However, methanogens synthesize high levels of coenzyme M, which has potential to act as a general antioxidant similar to glutathione. In support of this hypothesis, it has been shown that CoM can replace glutathione as the substrate in glutaredoxin-like oxidative stress enzymes from M. acetivorans and humans (50, 51). We observed that deletion of *ΔΔhdrABC* protects cells from molecular oxygen, and overproduction of CoM-SH protects cells from H_2_O_2_ stress. When combined, the *ΔhdrABC com^+^* strain can withstand exposure to 5% O_2_ or up to 2 mM H_2_O_2_ in oxidative stress, which is highly tolerant to oxidative stress relative to other anaerobes (52).

Molecular oxygen readily oxidizes flavins or labile organometallic cofactors, which are abundant in methanogens such as in heme cytochromes, coenzyme Ni(I)F430 in methyl coenzyme M reductase, Co(I/II)rrinoids in methyltransferases, and iron-sulfur clusters, resulting in generation of superoxide radical and inactivation of enzymes, loss of Fe(II) from Fes/S clusters, and subsequent damage to proteins, DNA, and lipid membranes (53). Superoxide is converted to H_2_O_2_ by superoxide dismutase (*sod*), of which there are two homologs in *M. acetivorans* (*MA_RS08180/MA1574*, and *MA_RS12570/MA2422*) through a GSH-independent process. H_2_O_2_ can then react with organometallic cofactors or free Fe(II) by Fenton chemistry to produce peroxide radicals, which readily react with sulfhydryls and iron-sulfur clusters; thus, exposure to O_2_ or H_2_O_2_ affects many enzymes involved in central metabolism. Cells may use catalase, in the form of *katE* or the bifunctional *katG* catalase/peroxidase, to convert H_2_O_2_ to water and O_2_, or anaerobic microbes may employ alternative cytochrome or rubredoxin systems to detoxify O_2_ or repair peroxide damage without producing O_2_ as a byproduct (52).

Previous investigators noted that *M. acetivorans* has an inactivating frameshift in *katE* (MA2081) but does seem to have an intact *katG* (MA0972) (31). Although cells do not have constitutive or inducible catalase activity, expression of *E. coli katG* (with heme supplementation) conferred increased resistance to H_2_O_2,_ but not to O_2_ (31). After prolonged selection with O_2_, *M. acetivorans* evolved resistance up to 2% O_2_, which correlated with increased expression of stress resistance genes (*sod, katG*, and alkyl peroxidase *apx*) and higher intracellular amounts of cysteine, CoM-SH, sulfide, and polyphosphate (46). We observed roughly equivalent resistance in the H_2_O_2_ andO_2_ stress assays for the *ΔhdrABC* and *ΔhdrABC com^+^* strains as when *E. coli katG* is introduced but without induction of *sod*, *katG,* or other predicted peroxidases (24), and the *ΔhdrABC com^+^* strain did not accumulate cysteine or sulfide and did not show a tendency to form biofilms (46).

We interpret these observations to suggest increased turnover of CoM and increased expression of corrinoid methyltransferases (24), especially with overexpression of *com* genes, may help scavenge reactive oxygen species to protect the cell from damage. These results also suggest that *M. acetivorans* could potentially be selected or engineered to resist higher levels of oxidative stress, for instance, by combining *ΔhdrABC com^+^* mutations with enhanced *katG* expression, upregulating peroxidases and cytochromes, or exploring fermentation process and stress conditions similar to approaches taken by others to enhance GSH production in yeast (54). Air-tolerant methanogens such as these could be desirable to enhance renewable biogas production or may enable using *M. acetivorans* for inexpensive synthesis of a wider array of chemicals that require molecular oxygen for biocatalysis such as taxol (paclitaxel) (55, 56).

## Data Availability

All data used to produce the figures and tables are provided in the manuscript.
